# A single serine to alanine substitution decreases bicarbonate affinity of phospho*enol*pyruvate carboxylase in C_4_*Flaveria trinervia*

**DOI:** 10.1093/jxb/ery403

**Published:** 2018-12-04

**Authors:** Robert J DiMario, Asaph B Cousins

**Affiliations:** School of Biological Sciences, Washington State University, Pullman, WA, USA

**Keywords:** Bicarbonate kinetics, C_4_ photosynthesis, membrane-inlet mass spectrometery, phospho*enol*pyruvate carboxylase

## Abstract

Phospho*enol*pyruvate (PEP) carboxylase (PEPc) catalyzes the first committed step of C_4_ photosynthesis generating oxaloacetate from bicarbonate (HCO_3_^−^) and PEP. It is hypothesized that PEPc affinity for HCO_3_^−^ has undergone selective pressure for a lower *K*_HCO3_ (*K*_m_ for HCO_3_^−^) to increase the carbon flux entering the C_4_ cycle, particularly during conditions that limit CO_2_ availability. However, the decrease in *K*_HCO3_ has been hypothesized to cause an unavoidable increase in *K*_PEP_ (*K*_m_ for PEP). Therefore, the amino acid residue S774 in the C_4_ enzyme, which has been shown to increase *K*_PEP_, should lead to a decrease in *K*_HCO3_. Several studies reported the effect S774 has on *K*_PEP_; however, the influence of this amino acid substitution on *K*_HCO3_ has not been tested. To test these hypotheses, membrane-inlet mass spectrometry (MIMS) was used to measure the *K*_HCO3_ of the photosynthetic PEPc from the C_4_*Flaveria trinervia* and the non-photosynthetic PEPc from the C_3_*F. pringlei*. The cDNAs for these enzymes were overexpressed and purified from the PEPc-less *PCR1 Escherichia coli* strain. Our work in comparison with previous reports suggests that *K*_HCO3_ and *K*_PEP_ are linked by specific amino acids, such as S774; however, these kinetic parameters respond differently to the tested allosteric regulators, malate and glucose-6-phosphate.

## Teaser

*Flaveria trinervia* C_4_ phospho*enol*pyruvate carboxylase (PEPc) has higher affinity for HCO_3_^−^ than its closely related *Flaveria pringlei* C_3_ PEPc, which drives higher modeled rates of C_4_ photosynthesis under low CO_2_ partial pressures.

## Introduction

Phospho*enol*pyruvate (PEP) carboxylase (PEPc) catalyzes the irreversible carboxylation of PEP using bicarbonate (HCO_3_^−^) to form the four-carbon sugar, oxaloacetate (OAA). In plants, this reaction generally influences stomatal conductance (Parvathi and Raghavendra, 1997; Cousins *et al.*, 2007), seed development (Sangwan *et al.*, 1992; O’Leary *et al.*, 2011), pH regulation (Davies, 1986; Britto and Kronzucker, 2005), and the balance between carbon and nitrogen metabolism by providing intermediates for the tricarboxylic acid (TCA) cycle (Rademacher *et al.*, 2002; Plaxton and Podestá, 2006). In mesophyll cells of C_4_ plants, PEPc catalyzes the first committed step of C_4_ photosynthesis by providing OAA that is subsequently modified to other four-carbon compounds before entering bundle sheath cells for decarboxylation, releasing CO_2_ at the site of Rubisco (Hatch *et al.*, 1975; von Caemmerer and Furbank, 2003).

Higher plants contain multiple PEPc-encoding (*ppc*) genes comprising a multigene family where most of the genes encode a non-photosynthetic C_3_ PEPc (Christin and Besnard, 2009). C_4_ plants obtained a modified PEPc isoform to power C_4_ photosynthesis through mutations to a native *ppc* coding region (Christin *et al.*, 2007; Rosnow *et al.*, 2014) and upstream promoter region (Schaffner and Sheen, 1992; Gowik *et al.*, 2004). Changes to the C_4_*ppc* promoter region led to strong, mesophyll-specific expression, resulting in high PEPc activity to drive the CO_2_-concentrating mechanism of C_4_ photosynthesis (Gowik *et al.*, 2004). Work on PEPc peptide sequences from members of the Poaceae, Amaranthaceae, Asteraceae, and Cyperaceae families (Christin *et al.*, 2007), as well as the Chenopodaceae family (Rosnow *et al.*, 2014), identified amino acid residues predicted to be under positive selection in these C_4_ lineages. Comparing PEPc sequences of species within and between families shows that C_4_ PEPc isoforms from different species possess different combinations of amino acid residues under positive selection (Christin *et al.*, 2007; Rosnow *et al.*, 2014). These findings suggest that there are multiple ways the C_4_ PEPc kinetic properties can arise in different C_4_ origins or that there is diversity in the PEPc kinetics between species.

An increase in PEPc activity in the leaf mesophyll cytosol in the intermediate C_3_/C_4_ species would be likely to lead to selection for changes in kinetic properties (Westhoff and Gowik, 2004). This was previously tested in a variety of C_3_/C_4_ species that displayed a progression in altered *K*_PEP_ (*K*_m_ for PEP) and decreased malate sensitivity (Westhoff and Gowik, 2004). C_4_ plants contain high levels of malate in the mesophyll cytosol, so there would be selection for amino acid substitutions that transition the malate-sensitive C_3_ PEPc to a less sensitive C_4_ PEPc (Bläsing *et al.*, 2002; Paulus *et al.*, 2013). This is supported by the Gly884 substitution in the *Flaveria trinervia* C_4_ PEPc to the *Flaveria pringlei* C_3_ PEPc arginine that caused the C_4_ PEPc to lose its resistance to malate (Paulus *et al.*, 2013).

As PEPc transitioned from C_3_ to C_4_ function, it has been suggested that certain amino acid substitutions were under positive selection to alter *K*_PEP_ and *K*_HCO3_ (*K*_m_ for HCO_3_^−^). It is hypothesized that certain mutations in the C_4_*ppc* coding region resulted from strong selective pressures to obtain a lower *K*_HCO3_ than that of the C_3_ PEPc (Jacobs *et al.*, 2008). The lower *K*_HCO3_ of the C_4_ PEPc may enhance the efficiency of C_4_ photosynthesis, especially when HCO_3_^−^ availably is low due to reduced stomatal conductance. Alternatively, the C_4_ PEPc has been shown to have a higher *K*_PEP_, with values typically reported between 100 µM and 590 µM (Svensson *et al.*, 1997; Dong *et al.*, 1998; Westhoff and Gowik, 2004; Lara *et al.*, 2006; Rosnow *et al.*, 2015), as compared with C_3_ non-photosynthetic PEPc *K*_PEP_ values which range from 13 µM to 60 µM (Westhoff *et al.*, 1997; Bläsing *et al.*, 2002; Gowik *et al.*, 2006; Lara *et al.*, 2006; Rosnow *et al.*, 2015). It was hypothesized that this increase in C_4_*K*_PEP_ was an unavoidable consequence of the reduction of *K*_HCO3_ since the two kinetic traits may be linked by certain amino acids (Jacobs *et al.*, 2008; Gowik and Westhoff, 2011). Alternatively, since the PEP pools in a C_4_ leaf are higher than in a C_3_ leaf, the high *K*_PEP_ of the C_4_ PEPc may ensure stronger diurnal regulation of PEPc (Budde and Chollet, 1986; Hatch, 1987).

Residue S774 in *F*. *trinervia* (S780 in maize) was shown to be under positive selection by Poetsch *et al.* (1991) and Hermans and Westhoff (1992), and substituting the conserved C_4_ serine for the conserved C_3_ alanine in *F*. *trinervia* (S774A) significantly decreased the *K*_PEP_ of the C_4_ PEPc (Bläsing *et al.*, 2000; Engelmann *et al.*, 2002). Since the S774A substitution affects the *K*_PEP_ of PEPc, it is possible that it may also affect the *K*_HCO3_, making S774 one of the residues potentially linking *K*_PEP_ and *K*_HCO3_. However, this serine residue was unimportant for the high *K*_PEP_ in the C_4_ Chenopodaceae (Rosnow *et al.*, 2014).

The only study to publish *K*_HCO3_ of both a C_3_ and C_4_ PEPc showed that the *K*_HCO3_ of five C_4_ species representing the Poaceae and Amaranthaceae families was ~26 µM compared with preliminary evidence suggesting that the *K*_HCO3_ of the C_3_ PEPc from *Flaveria cronquistii* (Asteraceae family) was 80 µM (Bauwe, 1986). Other studies reported C_4_ PEPc *K*_HCO3_ values ranging from 14 µM to 180 µM (Janc *et al.*, 1992; Gao and Woo, 1995; Parvathi *et al.*, 2000; Boyd *et al.*, 2015), where the C_3_*K*_HCO3_ of 80 µM falls within this reported range of C_4_*K*_HCO3_ values. However, comparing *K*_HCO3_ for closely related C_3_ and C_4_ PEPc isoforms can provide a more accurate analysis of the change in C_3_ to C_4_*K*_HCO3_ and whether there was a strong selective force on PEPc *K*_HCO3_, but to date this has not been performed. Additionally, the previously reported *K*_HCO3_ values were obtained by coupling PEPc activity to spectrophotometrically measured NADH oxidation rates. It is difficult to obtain accurate *K*_HCO3_ values using this method because it does not directly measure changes in HCO_3_^−^ concentration in the assay. This is problematic because measurements of *K*_HCO3_ require accurate determinations of PEPc activity and HCO_3_^−^ concentrations below the *K*_HCO3_, which is in the micromolar range. To overcome this problem, membrane-inlet mass spectrometry (MIMS) can be used to measure HCO_3_^−^ consumption by PEPc accurately and directly in real-time over a wide range of inorganic carbon (C_i_) concentrations, including concentrations well below the *K*_HCO3_, without the complication of coupling PEPc activity to the NADH dehydrogenase reaction (Boyd *et al.*, 2015).

In this study, we use MIMS to obtain *K*_HCO3_ values for the photosynthetic PEPc from the C_4_ plant *F*. *trinervia* and the non-photosynthetic PEPc from the C_3_ plant *F. pringlei* that were overexpressed and purified from the PEPc-less *PCR1 Escherichia coli* strain (Sabe *et al.*, 1984; Svensson *et al.*, 1997). We found that the S774A substitution increases the C_4_*K*_HCO3_, whereas the A774S substitution did not affect the C_3_*K*_HCO3_, suggesting that additional amino acids besides S774 are involved in the C_4_*K*_HCO3_ trait. Since previous studies reported PEPc *K*_PEP_ changing in the presence of the allosteric activator glucose 6-phosphate (G6-P) and the inhibitor malate (Huber and Edwards, 1975; Wedding *et al.*, 1990; Gupta *et al.*, 1994; Bläsing *et al.*, 2002), we tested whether these allosteric regulators also affected *K*_HCO3_. We report that G6-P and malate have a minimal effect on the *K*_HCO3_ of PEPc. We address how differences in calibration methods, assay conditions, and enzyme extractions can produce different *in vitro* kinetic values, and we report an improvement to the MIMS PEPc assay. Lastly, we demonstrate how the decrease in *K*_HCO3_ between the C_3_ and C_4_ PEPc isoforms increases the modeled rates of C_4_ photosynthesis at low CO_2_ concentrations.

## Materials and methods

### Generating *PCR1* PEPc-overexpressing lines

The PEPc-less *E. coli* strain, *PCR1* (Sabe *et al.*, 1984), and PEPc cDNA constructs used in Svensson *et al.* (1997) and Bläsing *et al.* (2000) were generously provided by Professor Peter Westhoff’s lab. The plasmid, pTrc99A, carrying the cDNA coding for either the C_4_*F. trinervia* PEPc, the C_3_*F. pringlei* PEPc, or *Flaveria* PEPc with either an alanine or serine substitution at residue 774, C_4_-S774A or C_3_-A774S, respectively, was transformed into the *PCR1 E*. *coli* strain. *PCR1* transformants producing plant PEPc were selected following the method of Svensson *et al.* (1997).

### Growth of *PCR1* PEPc-overexpressing lines for PEPc extraction

A 4 ml growth culture (Luria–Bertani broth; 0.1% w/v dextrose; 100 µg ml^−1^ ampicillin) was inoculated with a glycerol stock of the *PCR1* strain carrying a *Flaveria* PEPc construct and was incubated at 28 °C with shaking at 160 rpm overnight. The following morning, the 4 ml culture was centrifuged at 1538 *g* for 10 min at room temperature. The supernatant was discarded, and the bacterial pellets were resuspended and transferred to a large 500 ml growth culture which was incubated at 28 °C and shaken at 160 rpm. After 6 h of incubation, isopropyl-β-d-1-thiogalactopyranoside (IPTG) was added to a final concentration of 100 µM to the 500 ml growth culture to induce PEPc production overnight.

### PEPc extraction and purification from *E*. *coli*

The 500 ml growth culture was centrifuged at 2602 *g* for 10 min at room temperature and the bacterial pellets were resuspended in a total volume of 20 ml of ice-cold lysis buffer [50 mM Tris–HCl, pH 8.0; 0.5 M NaCl; 10 mM DTT; 1 mM EDTA, pH 8.0; 20 µl ml^−1^*E*. *coli* protease inhibitor (Sigma); 1 mg ml^−1^ lysozyme (Bioworld); 10% (v/v) glycerol; 20% (w/v) sucrose]. The resuspended cells were placed in ice for 30 min and then lysed via sonication (BioLogics Ultrasonic Homogenizer 300 V/T). The sonicated cells were transferred to centrifuge tubes and were spun at 30597 *g* for 30 min at 4 °C. The supernatant was collected and MgCl_2_ was added to the supernatant to a final concentration of 10 mM. Polyethylene glycol (50% PEG 8000) was added to the supernatant to a final concentration of 6% (v/v) before placing the supernatant on ice for 15 min with gentle mixing. The supernatant was again spun at 30597 *g* for 20 min at 4 °C. The protein pellets were discarded and 50% PEG 8000 was added to the final concentration of 12% (v/v). The supernatant was slowly stirred on ice for 15 min before centrifugation at 30597 *g* for 20 min at 4 °C.

The protein pellet was collected and resuspended in 6 ml of Buffer A [0.5 M (NH_4_)_2_SO_4_; 20 mM Tris–HCl, pH 7.5; 0.1 mM DTT; 1 mM EDTA, pH 8.0; 5% (v/v) glycerol] supplemented with 1 mM phenylmethylsulfonyl fluoride (PMSF) (Svensson *et al.*, 1997). The protein sample was loaded onto a HIC phenyl–Sepharose column (1 cm×5.5 cm) pre-incubated with Buffer A at a flow rate of 1.5 ml min^−1^. The hydrophobic properties of PEPc were used for the partial purification of PEPc by following the protocol of Svensson *et al.* (1997).

Fractions from the phenyl–Sepharose column were analyzed for PEPc activity by coupling the PEPc and NADH dehydrogenase reactions following the protocol of Boyd *et al.* (2015). Fractions displaying the highest PEPc activity were pooled and desalted in Buffer B [100 mM HEPES-KOH, pH 7.6; 1 mM DTT; 1 mM EDTA, pH 8.0] and concentrated using Corning Spin-X UF columns (6 ml volume, 100 K molecular weight cut-off) according to the Corning procedure. Glycerol was added to a final concentration of 20% (v/v) before storage at –80 °C. Total protein content of the PEPc samples was measured by a modified Bradford assay (Bio-Rad Protein Assay Kit II) (Bradford, 1976) using the Bio-Rad procedure.

### Extracting PEPc from *F. trinervia* and *Setaria viridis*

PEPc samples were extracted and desalted from leaves of *F. trinervia* and *S. viridis* following the procedure of Boyd *et al.* (2015). Once the desalted PEPc extracts were collected and moved through a Millex-GP 0.22 µm syringe filter (Millipore), the extracts were concentrated by spinning the samples at 2880 *g* for 20 min at 4 °C in an Amicon Ultra-4 Ultracel-100K centrifugal filter (Millipore). Glycerol was added to the concentrated PEPc samples to a final concentration of 20% (v/v) and stored at –80 °C.

### Obtaining *V*_Pmax_, *K*_HCO3_, and Hill values for the different PEPc isoforms

The HCO_3_^−^-dependent PEPc assays were run in a 600 µl cuvette attached to the inlet of a mass spectrometer as described by Cousins *et al.* (2010). A CO_2_ calibration was conducted before each HCO_3_^−^ response curve as reported by Boyd *et al.* (2015). The calibration consisted of three 2 µl injections of 10 mM NaHCO_3_ into 0.1 N HCl and three 6 µl injections of 100 mM NaHCO_3_ into the PEPc reaction mixture [100 mM HEPES-KOH, pH 7.6; 10 mM MgCl_2_; 1 mM DTT; 50 µg ml^−1^ carbonic anhydrase (CA); 5 mM G6-P; 5 mM PEP]. The second calibration step differs from Boyd *et al.* (2015) as their 100 mM NaHCO_3_ injections went into buffer lacking DTT, G6-P, and PEP. DTT, G6-P, and PEP were added to the second calibration to take into account the pH changes these compounds have on the assay buffer. As the CO_2_ calibrations take into account total C_i_ and total CO_2_ in the cuvette, the HCO_3_^−^ concentration could be deduced by subtracting the amount of CO_2_ in the cuvette from the total C_i_ measured in the cuvette (Boyd *et al.*, 2015).

To measure PEPc HCO_3_^−^ kinetics, seven NaHCO_3_ concentrations (50, 100, 200, 350, 500, 750, and 1000 µM) were used for the assays. The CO_2_ was removed from the assay buffer containing 100 mM HEPES-KOH, pH 7.6 and 10 mM MgCl_2_ by continuously bubbling the buffer with humidified N_2_ gas starting at least 1 h prior to initiating the assays. The assay buffer (600 µl) followed by 1 mM DTT, 50 µg ml^−1^ CA, 5 mM G6-P, 5 mM PEP, and various NaHCO_3_ concentrations were added to the reaction cuvette and held at a constant 25 °C with a temperature-controlled water bath.

A blank rate was obtained by measuring the change in the mass 44 (^12^C^16^O^16^O) signal during a 30 s period before initiating the reaction with the addition of 10–15 µg of total protein of the PEPc extract. The PEPc reaction was run for 5 min but the first 20 s of the PEPc reaction were discarded to allow for enzyme mixing and rate stabilization. The MIMS reports a mass 44 signal every 0.8 s, so a robust 10 s running average of the change in mass 44 was used as a single data point for PEPc activity (*V*_P_) at an averaged [HCO_3_^−^]. Data points from 10 s running averages were taken immediately following the 20 s mixing phase. For the larger NaHCO_3_ injections (350–1000 µM NaHCO_3_), 10 s running averages were taken until a drop in PEPc activity was observed to avoid data points where there might be end-product inhibition of the reaction. For the lower NaHCO_3_ concentrations (50–200 µM), 10 s running averages were taken until the reaction was depleted of C_i_. Once the C_i_ was depleted from the 50, 100, and 200 µM NaHCO_3_ injections, as indicated by a zero slope for the mass 44 signal, a 30 s running average of the zero slope was taken to obtain a mass 44 zero. These mass 44 zeroes accounted for mechanical drift in the MIMS as the different zeroes were taken at various times throughout the HCO_3_^−^ response curve.

The kinetic parameters *V*_Pmax_, *K*_HCO3_, and Hill value (*h*) were obtained by using the Hill equation:

$$V_{P}=\frac{V_{Pmax}\;\times \;{\left[{\text{HCO}}_{3}^{-}\right]}^{\text{h}}}{{\left(K_{HCO3}\right)}^{\text{h}}\;+\;{\left[{\text{HCO}}_{3}^{-}\right]}^{\text{h}}}$$ (1)

where the Hill equation was fit to the HCO_3_^−^ response curve using Excel’s Solver function to produce the kinetic parameters listed above.

### Measuring the impact of G6-P and malate on *K*_HCO3_

To measure the effect of G6-P on *K*_HCO3_, G6-P was omitted from the assay described above to compare *K*_HCO3_ values in the presence or absence of 5 mM G6-P. Alternatively, 2.5 mM malate (pH 7.6) was added to the assay described above to determine if malate affects PEPc *K*_HCO3_ values in the presence or absence of G6-P.

### Measuring PEP effects on malate inhibition

The MIMS assay described above was used to determine if PEP concentration affects malate inhibition of PEPc, in the absence of G6-P. Five malate concentrations were used (0, 1, 2, 3, and 4 mM) to determine the percentage change in enzyme activity of the C_3_ and C_4_ PEPc isoforms in the presence of saturating NaHCO_3_ (1000 µM) and when PEP was saturating (5 mM) or non-saturating (150 µM and 750 µM for the C_3_ and C_4_ isoforms, respectively). The non-saturating PEP concentrations were determined to be twice the reported *K*_PEP_ values (2×*K*_PEP_) in the absence of G6-P (Westhoff and Gowik, 2004).

### Extraction source, pH, and calibration effects on PEPc *K*_HCO3_

Previously, MIMS measurements of desalted PEPc extracts from *S. viridis* reported a *K*_HCO3_ of 62.8 ± 5.0 µM (Boyd *et al.*, 2015). Therefore, we tested whether differences in PEPc source (plant versus *E*. *coli*), pH, or MIMS calibrations caused the *K*_HCO3_ reported here to differ from those of Boyd *et al.* (2015). The MIMS assay described above containing 5 mM G6-P and no malate was used to test whether PEPc samples extracted from leaves of *F. trinervia* and *S. viridis* produced different *K*_HCO3_ values from the C_4_ PEPc extracted from *E*. *coli*. For each assay, 5–10 µl of plant extract was added to initiate the PEPc reaction. PEPc extracts from *S*. *viridis* were used to compare *K*_HCO3_ values obtained at pH 7.6, the pH used in this study, with *K*_HCO3_ values obtained at pH 7.8 used by Boyd *et al.* (2015). These extracts were also used to compare *K*_HCO3_ values obtained at pH 7.8 using either the current calibration method outlined above or the calibration method of Boyd *et al.* (2015).

### Statistical analysis of experimental data

Statistical analyses of the kinetic data were performed using RStudio version 1.1.447 (RStudio Team, 2016). Homogeneity of variance was checked using Levene tests, and normality was checked using residual quantile plots and residual versus fitted value plots. Non-normal data were log transformed but they reported the same statistical outcomes as non-transformed data, so for simplicity only non-transformed data analyses are presented. One-way ANOVA and Tukey HSD post-hoc tests were used to determine statistical significance (*P*<0.05) of *K*_HCO3_ between PEPc isoforms. Two-way ANOVA (*P*<0.05) and Tukey HSD post-hoc tests were used to analyze statistically significant differences in *K*_HCO3_ between isoforms and the impact of potential allosteric effectors. A two-way repeated measures ANOVA (*P*<0.05) was used to test if the change in PEPc activity in response to malate significantly differed between C_3_ and C_4_ PEPc isoforms at various PEP concentrations. One-way ANOVA and Tukey HSD post-hoc tests were used to determine significant differences between C_4_ PEPc extracted from *E*. *coli* and PEPc extracted from *F*. *trinervia* and *S*. *viridis* leaves. Student’s *t*-tests (*P*<0.05) were separately used to determine significant differences in *S*. *viridis* PEPc *K*_HCO3_ assayed at pH 7.6 and 7.8 and for *S*. *viridis* PEPc *K*_HCO3_ assayed at pH 7.8 using the two calibration methods.

### Modeling the effect of *K*_HCO3_ on C_4_ photosynthesis

The modeled effect of *K*_HCO3_ on the response of C_4_ enzyme-limited photosynthetic CO_2_ assimilation (*A*_c_) to changing mesophyll CO_2_ concentrations (C_m_) was determined by solving the quadratic formula using the set of equations as described by von Caemmerer (2000). The equations and input variables were taken from von Caemmerer *et al.* (1994), von Caemmerer (2000), Tholen and Zhu (2011), and Ubierna *et al.* (2013), and are presented in Supplementary Table S1 at *JXB* online.

## Results

### Kinetics of the C_3_, C_4_, and chimeric PEPc isoforms

**Fig. 1.**
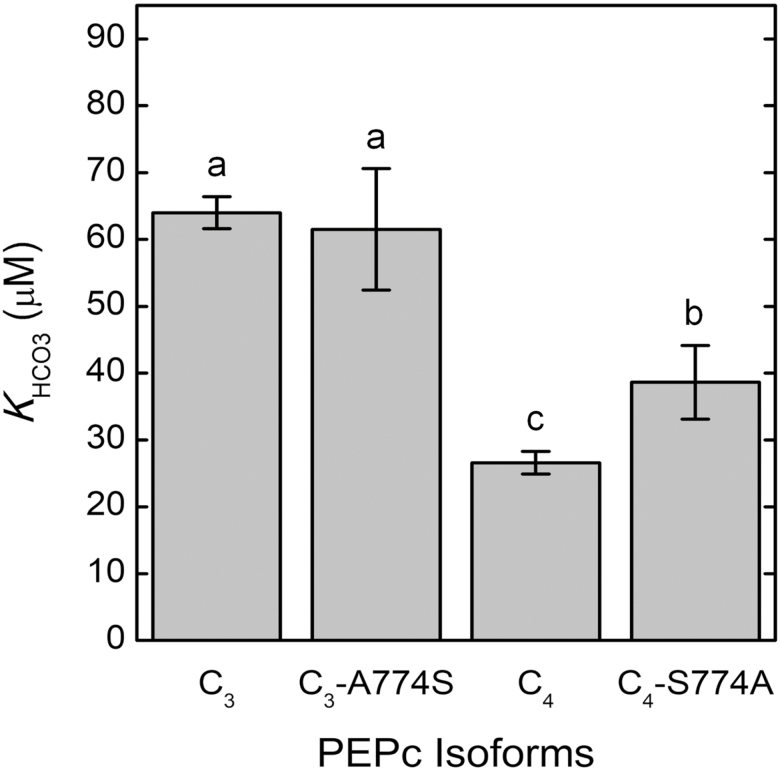
The *K*_HCO3_ of the C_3_, C_4_, and chimeric PEPc isoforms. The *K*_HCO3_ values were obtained from the MIMS assayed in 100 mM HEPES-KOH buffer (pH 7.6) that contained 10 mM MgCl_2_, 5 mM PEP, 50 µg ml^−1^ CA, 1 mM DTT, and 5 mM G6-P. Error bars represent the mean ±SD of four independent extractions from *E*. *coli* for each PEPc isoform. Significance was determined by one-way ANOVA and Tukey HSD post-hoc tests. Bars with different letters are significantly different (*P*<0.05).

The Hill equation was used to determine the maximum rate of PEPc carboxylation (*V*_Pmax_), the *K*_m_ for bicarbonate (*K*_HCO3_), and the co-operativity of the PEPc active sites (*h*) from 25 °C MIMS measurements of PEPc activity (*V*_p_) in response to changes in HCO_3_^−^ concentrations (Supplementary Fig. S1). Measurements were made on C_4_, C_3_, and chimeric *Flaveria* PEPc isoforms expressed and partially purified from *E. coli*. The C_4_ PEPc had a significantly lower *K*_HCO3_ than the C_3_ PEPc, 26.6 ± 1.7 µM and 64.0 ± 2.4 µM, respectively (Fig. 1). Additionally, the C_3_ PEPc had a lower *V*_Pmax_ compared with the C_4_ PEPc, 5.1 ± 0.7 µmol mg protein^−1^ min^−1^ and 8.1 ± 0.7 µmol mg protein^−1^ min^−1^, respectively (Supplementary Table S2). Neither isoform displayed co-operativity towards HCO_3_^−^ binding, with Hill values close to 1.0 under all assay conditions (Supplementary Table S2).

The substitution of the conserved C_4_ serine at residue 774 (780 in maize) with the conserved C_3_ alanine (C_4_-S774A) significantly increased the *K*_HCO3_ by 45% from 26.6 ± 1.7 µM to 38.6 ± 5.5 µM (Fig. 1). However, the C_4_-S774A substitution had no effect on *V*_Pmax_ (Supplementary Table S2). The reverse substitution, C_3_-A774S made in the C_3_ PEPc, did not significantly change the *K*_HCO3_ (from 64.0 ± 2.4 µM to 61.5 ± 9.1 µM; Fig. 1) nor did it affect *V*_Pmax_ (Supplementary Table S2).

### The impact of G6-P and malate on *K*_HCO3_

**Fig. 2.**
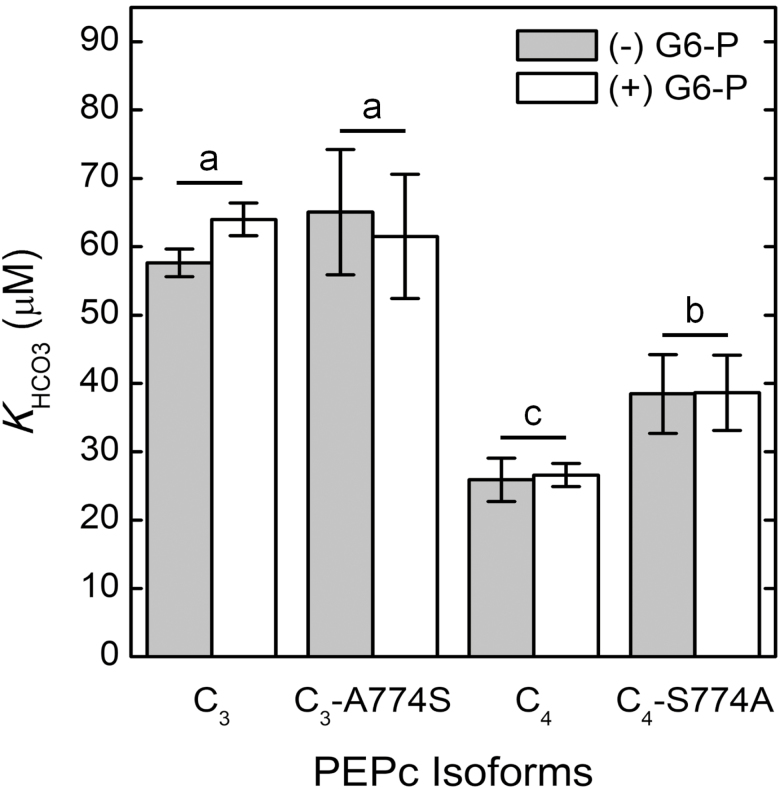
The impact of G6-P on the *K*_HCO3_ of the different PEPc isoforms. The *K*_HCO3_ values of the different PEPc isoforms were obtained from MIMS PEPc assays where 5 mM G6-P was present (white bars) or absent (gray bars) in the assay buffer. White bars are data represented from Fig. 1. Error bars represent the mean ±SD of four independent extractions from *E. coli* for each PEPc isoform. A two-way ANOVA determined that G6-P had a non-significant effect on *K*_HCO3_ but there was a significant isoform effect, and a Tukey HSD post-hoc test was used to determined significance. PEPc isoforms with different letters are significantly different (*P*<0.005).

The *V*_Pmax_ did not change by omitting G6-P from the assay, regardless of the PEPc isoform (Supplementary Table S2). Additionally, the *K*_HCO3_ values of the PEPc isoforms were not significantly altered by the presence or absence of G6-P in the assay (Fig. 2).

**Fig. 3.**
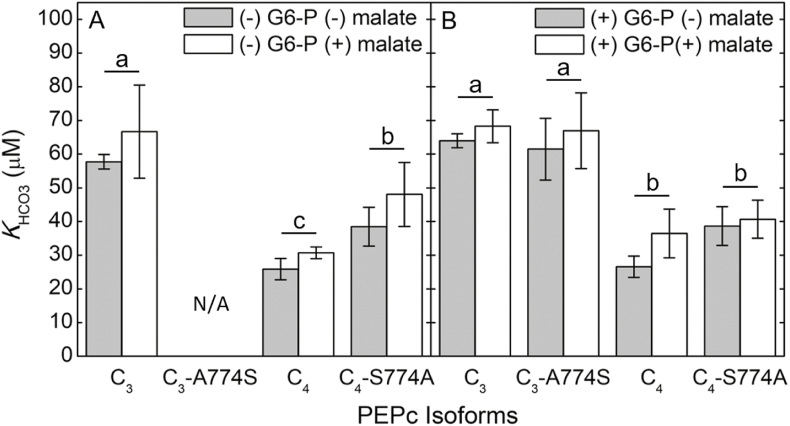
The effect of 2.5 mM malate on the *K*_HCO3_ of different PEPc isoforms in the absence and presence of G6-P. (A) *K*_HCO3_ values of the different PEPc isoforms obtained from MIMS PEPc assays in the presence (white bars) or absence (gray bars) of 2.5 mM malate without G6-P. (B) The *K*_HCO3_ of the different PEPc isoforms with 5 mM G6-P, in the presence (white bars) or absence (gray bars) of 2.5 mM malate. Gray bars in both (A) and (B) represent data from previous figures. Error bars represent the mean ±SD of four independent extractions from *E. coli* for each PEPc isoform. A two-way ANOVA determined significant effects on *K*_HCO3_ by allosteric regulators and between different isoforms, but a Tukey HSD post-hoc test determined no significant allosteric effect on *K*_HCO3_ in both (A) and (B). A Tukey HSD post-hoc test was performed on the isoform effect, and different letters represent significant differences (*P*<0.005).

**Fig. 4.**
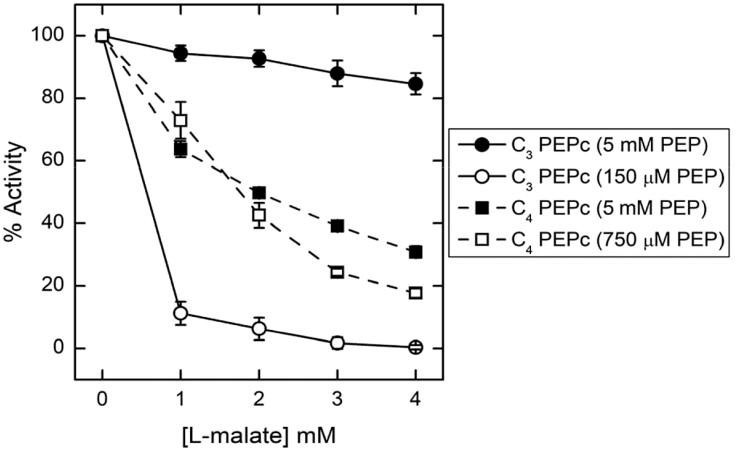
Malate resistance of the C_3_ PEPc is affected more by changing PEP concentrations than that of the C_4_ PEPc. At 5 mM PEP (filled circles and squares), the C_3_ PEPc (solid line) is more resistant to malate than the C_4_ PEPc (dashed line). When the PEP concentration was dropped to twice the reported *K*_PEP_ for the C_3_ and C_4_ PEPc isoforms (open circles and squares), malate resistance of the C_3_ PEPc dropped drastically compared with the malate resistance for the C_4_ PEPc. Malate activity assays were performed in 100 mM HEPES-KOH (pH 7.6), 10 mM MgCl_2_, 1 mM DTT, 50 µg ml^−1^ CA, 2.5 mM malate (pH 7.6), 1 mM NaHCO_3_, and various PEP concentrations. Shapes and error bars represent the mean ±SD of four independent extractions from *E*. *coli* for both the C_3_ and C_4_ PEPc isoforms. A two-way repeated measures ANOVA (*P*<0.05) determined that there was a significant difference between the increased sensitivity to malate of the C_3_ PEPc versus the C_4_ PEPc when PEP concentration was decreased to 2×*K*_PEP_ for each isoform.

Under the current measurement conditions of pH 7.6, 5 mM PEP, and the absence of G6-P, the addition of 2.5 mM malate decreased the *V*_Pmax_ in the C_4_, C_4_-S774A chimeric, and the C_3_ PEPc by 57.5, 24.4, and 6.8%, respectively (Supplementary Tables S2, S3). However, the *K*_HCO3_ values of the PEPc isoforms were not significantly altered by the presence of malate in the assay (Fig. 3A). HCO_3_^−^ response curves in the absence of G6-P were not obtained for the C_3_-A774S PEPc due to severe inhibition of the chimeric PEPc by malate (Supplementary Fig. S2). When 5 mM G6-P and 2.5 mM malate were both present in the PEPc assay, *V*_Pmax_ decreased in the C_4_, C_4_-S774A chimeric, C_3_, and C_3_-A774S chimeric PEPc by 44.4, 28.6, 2, and 14%, respectively (Supplementary Tables S2, S3). However, the *K*_HCO3_ values of the PEPc isoforms were not significantly changed with both G6-P and malate in the assay (Fig. 3B). Interestingly, decreasing the total PEP concentration in the assay from 5 mM to twice the reported *K*_PEP_ of the C_3_ and C_4_ isoforms (Bläsing *et al.*, 2002; Paulus *et al.*, 2013), 150 µM and 750 µM PEP, respectively, caused the C_3_ PEPc to lose activity dramatically in the presence of malate, whereas the change in PEP concentration had a smaller effect on malate inhibition of C_4_ PEPc activity (Fig. 4).

### Effects of MIMS calibrations, assay conditions, and extraction sources on *K*_HCO3_

**Fig. 5.**
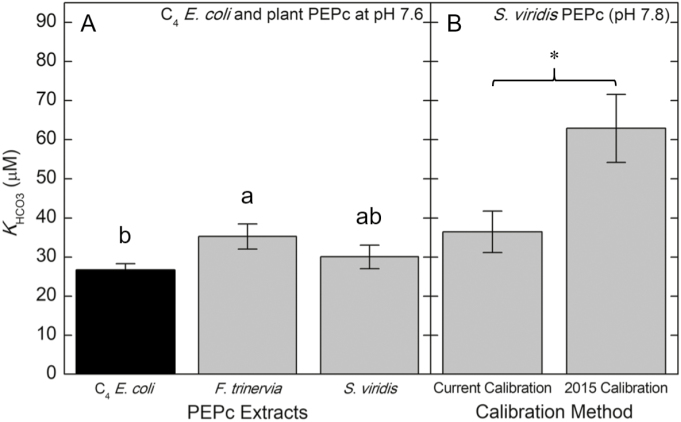
The *K*_HCO3_ measured under different assay conditions for PEPc isoforms extracted from *F. trinervia*, *S. viridis*, and *E. coli*. (A) The *K*_HCO3_ values of the C_4_ PEPc extracts from *E. coli* (black bar; value from Fig. 1) and desalted PEPc extracts from *F. trinervia* and *S. viridis* (gray bars) were obtained from the MIMS assayed in 100 mM HEPES-KOH buffer (pH 7.6) with 10 mM MgCl_2_, 5 mM PEP, 50 µg ml^−1^ CA, 1 mM DTT, and 5 mM G6-P. Bars represent the mean ±SD of four independent PEPc extractions. Significance was determined by one-way ANOVA and Tukey HSD tests. Bars with different letters are significantly different (*P*<0.05). (B) *S*. *viridis* PEPc *K*_HCO3_ values measured from assays at pH 7.8 using either the current MIMS calibration or the Boyd *et al.* (2015) calibration. Significance between the *K*_HCO3_ of *S. viridis* PEPc assayed at (A) pH 7.6 and (B) 7.8 was determined by a Student’s *t*-test (*P*=0.08). Significance between the *K*_HCO3_ of *S*. *viridis* PEPc assayed at pH 7.8 obtained by either the current calibration method or the Boyd *et al.* (2015) calibration method was determined by a Student’s *t*-test (*P*<0.05).

At pH 7.6, the C_4_*F. trinervia* PEPc extracted from *E. coli* had a lower *K*_HCO3_ than desalted plant PEPc extracts from *F. trinervia*, 26.6 ± 1.7 µM and 35.2 ± 3.2 µM, respectively (Fig. 5A). Although not statistically significant, changing the pH of the assay buffer from 7.6 to 7.8 increased the *K*_HCO3_ of *S. viridis* PEPc by 21.3% from 30.0 ± 3.0 µM to 36.4 ± 5.3 µM (Fig. 5A, 5B). When using the Boyd *et al.* (2015) MIMS calibration method at pH 7.8, the *S. viridis K*_HCO3_ increased to 62.9 ± 8.7 µM (Fig. 5B).

### Modeling the effect of different *K*_HCO3_ values on C_4_ photosynthesis

**Fig. 6.**
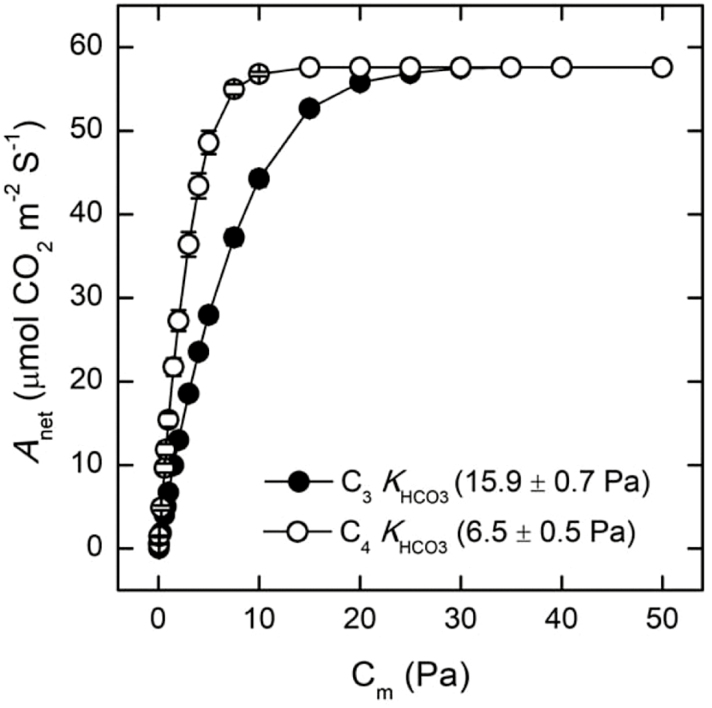
Modeled rates of C_4_ photosynthesis with C_3_ and C_4_*K*_HCO3_. The *K*_HCO3_ values for the C_3_ PEPc (filled circles) and C_4_ PEPc (open circles) were input into the C_4_ photosynthesis model from von Caemmerer (2000) to determine the modeled rate of CO_2_ assimilation (*A*_net_) at various mesophyll CO_2_ concentrations (C_m_). Symbols represent means ±SD of four independent *K*_HCO3_ values presented in Fig. 1 input into the model, where all other variables in the C_4_ model were held constant. The maximal rates of PEP regeneration (*V*_pr_), Rubisco carboxylation (*V*_Cmax_), and maximum PEPc carboxylation per unit leaf area [*V*_Pmax(plant)_] were set to 80, 60, and 120 µmol m^−2^ s^−1^, respectively (von Caemmerer, 2000), and all other values are presented in Supplementgary Table S1. A p*K*_a_ of 6.12 and assumed a mesophyll cytosol pH of 7.2 were used to convert µM HCO_3_^−^ to µM CO_2_. Pa CO_2_ was obtained by using Henry’s constant for CO_2_ (0.034 mol l^−1^ atm^−1^) and assumed standard pressure (101325 Pa atm^−1^).

The C_3_ and C_4_ PEPc *K*_HCO3_ values from Fig. 1 were input into the C_4_ photosynthesis model from von Caemmerer (2000) to determine how differences in *K*_HCO3_ would impact modeled rates of C_4_ photosynthesis. Varying *K*_HCO3_ with a constant *V*_Pmax_ significantly changed the modeled rates of C_4_ photosynthesis under CO_2_ conditions below 20 Pa. The lower *K*_HCO3_ of the C_4_ PEPc resulted in higher modeled rates of C_4_ photosynthesis at these low mesophyll CO_2_ concentrations (C_m_; Fig. 6). However, the difference between the C_3_ and C_4_*K*_HCO3_ modeled no differences in net CO_2_ assimilation above ~20 Pa C_m_ (Fig. 6).

## Discussion

### Kinetic changes during the evolution of the C_4_ PEPc

We and others (Jacobs *et al.*, 2008; Gowik and Westhoff, 2011) have hypothesized that there was a strong selective pressure to reduce the *K*_HCO3_ of the C_4_ PEPc isoform. Additionally, previous studies have reported that changing the PEPc amino acid residue 774 in *Flaveria* spp. (780 in maize) influences *K*_PEP_ and its allosteric regulation (Engelmann *et al.*, 2002; Endo *et al.*, 2008). Therefore, the aim of this research was to test the hypotheses that the *K*_HCO3_ of the C_4_ PEPc isoform from *F. trinervia* would be lower than the *K*_HCO3_ of the C_3_ PEPc isoform from *F. pringlei* and that changes to residue 774 will impact *K*_HCO3_ and its allosteric regulation. Residue 774 was chosen because others have shown the C_4_*F*. *trinervia* S774A substitution reduces *K*_PEP_ (Bläsing *et al.*, 2000; Endo *et al.*, 2008). Furthermore, residue 774 is near both the PEP- and HCO_3_^−^-binding sites, and may also influence *K*_HCO3_. We have analyzed the influence of this residue on *K*_HCO3_ and showed that the C_4_-S774A chimeric PEPc had a significantly higher *K*_HCO3_ compared with the C_4_ PEPc (Fig. 1). This fits with previous data that suggest that *K*_PEP_ and *K*_HCO3_ are inversely linked through specific amino acid residues near the two binding sites. For example, the K829G substitution in the *F. trinervia* C_4_ PEPc resulted in a small decrease in *K*_HCO3_ and a simultaneous increase to *K*_PEP_ (Gao and Woo, 1996). Alternatively, swapping Lys600 with either an arginine or threonine in *F. trinervia* led to increases in both *K*_PEP_ and *K*_HCO3_, but this residue is one of the four conserved amino acids comprising the HCO_3_^−^-binding site (Gao and Woo, 1995; Kai *et al.*, 2003).

The chimeric C_3_ PEPc of *F. pringlei*, C_3_-A774S, had a minimal effect on *K*_HCO3_ (Fig. 1). This same amino acid substitution was also shown not to influence the *K*_PEP_ of the C_3_ PEPc with G6-P present in the assay. However, in the absence of G6-P, the same A774S substitution did increase *K*_PEP_ (Bläsing *et al.*, 2000). Taken together, these results support the analysis that multiple amino acid residues, in addition to S774 (S780 in maize), influence PEPc kinetics. Indeed, swapping amino acid residues 296–437 from the C_4_ enzyme into the C_3_-A774S chimeric PEPc increased the C_3_*K*_PEP_ even closer to the established C_4_*K*_PEP_ value (Engelmann *et al.*, 2002). However, further research is needed to understand how changes in PEPc amino acid composition influence *K*_HCO3_, particularly how specific amino acid changes influence the impact that allosteric regulators such as G6-P and malate have on *K*_HCO3_.

### The influence of G6-P and malate on *K*_HCO3_

PEPc is regulated by post-translational modifications (PTMs) and interactions with allosteric effectors. Previous studies showed that glycine and G6-P activate PEPc (Svensson *et al.*, 1997; Westhoff *et al.*, 1997; Endo *et al.*, 2008) while the end-products, aspartic acid and malate, inhibit PEPc (Huber and Edwards, 1975). Additionally, previous studies have shown that the PEP-binding sites of the C_4_ PEPc tetramer display positive co-operativity for *K*_PEP_, which can be altered by the binding of G6-P and malate to PEPc (Wedding *et al.*, 1990; Bläsing *et al.*, 2002; Rosnow *et al.*, 2015). We show that neither G6-P nor malate appears to impact *K*_HCO3_ and the co-operativity of HCO_3_^−^ binding to the extent that they influence *K*_PEP_ and co-operative PEP binding (Figs 2, 3; Supplementary Tables S2, S3). Using the crystal structure (4BXC) deposited by Schlieper *et al.* (2014), the HCO_3_^−^-binding site is further from the G6-P and aspartic acid/malate allosteric binding sites than the PEP-binding site is from the allosteric sites, so any structural changes to PEPc caused by allosteric binding may affect the PEP-binding site more than the HCO_3_^−^-binding site. Additionally, the S774A and A774S substitutions did not influence the allosteric regulation of PEPc to the extent that the R884G and G884R substitutions affected malate sensitivity of the *F*. *pringlei* and *F*. *trinervia* PEPc isoforms, respectively (Paulus *et al.*, 2013). This discrepancy may be due to residue 884 being closer to the residues of the aspartate/malate-binding sites compared with residue 774, whereas residue 774 is closer to the PEP- and HCO_3_^−^-binding sites (Kai *et al.*, 2003; Paulus *et al.*, 2013).

Another possibility is that PEP binds before HCO_3_^−^ (Janc *et al.*, 1992), potentially conferring the primary allosteric regulation of PEPc to the binding of PEP. Alternatively, under our assay conditions, the high PEP concentration may have reduced the impact G6-P and malate had on *K*_HCO3_. For example, G6-P has a greater activating effect on PEPc under limiting PEP concentrations at 0.5 mM (Gupta *et al.*, 1994). In addition, multiple studies suggest that there is a regulatory PEP-binding site different from the PEPc active site (Rustin *et al.*, 1988; Rodríguez-Sotres and Muñoz-Clares, 1990; Mújica-Jiménez *et al.*, 1998; Yuan *et al.*, 2006), and it is possible this regulatory PEP site may not be saturated under low PEP concentrations. Saturating this regulatory PEP-binding site might supersede G6-P activation and overcome malate inhibition of PEPc (Huber and Edwards, 1975). The assay conditions used in this study contained saturating (5 mM) levels of PEP, which were well above the *K*_PEP_ of both PEPc isoforms, since limiting PEP would complicate the response to changes in HCO_3_^−^ concentrations. The C_3_ PEPc was more resistant to malate inhibition than the C_4_ PEPc under these saturating PEP conditions, which is in contrast to previous reports (Bläsing *et al.*, 2002; Paulus *et al.*, 2013). However, we found that the C_3_ PEPc was more sensitive to malate than the C_4_ PEPc when the PEP concentration in the assay was reduced to 2×*K*_PEP_ (Fig. 4). This suggests that the PEP regulatory site for the C_4_ PEPc may be less sensitive than the C_3_ PEPc to changes in free PEP availability. Alternatively, the C_4_ PEP regulatory site may not have as much influence on malate tolerance as the C_3_ PEP regulatory site under the current assay conditions.

We were unable to obtain kinetic data for the C_3_-A774S chimeric PEPc due to drastic inhibition of the enzyme by malate when G6-P was absent from the assay (Supplementary Fig. S2). This result was unexpected since the addition of 2.5 mM malate had a small effect on the activity of the C_3_ PEPc (Supplementary Tables S2, S3). However, since PEP and malate interact differently with the C_3_ and C_4_ PEPc isoforms, it is possible that the A774S substitution in the C_3_ PEPc modified these interactions to allow potent inhibition of the C_3_-A774S chimeric PEPc. Further analysis is needed to test the extent of malate inhibition on the C_3_-A774S PEPc and other chimeric PEPc isoforms under various assay conditions. It is worth noting that malate has a stronger inhibitory effect on PEPc at pH 7.0 than at pH 8.0 (Huber and Edwards, 1975; Gupta *et al.*, 1994). As discussed below, pH and other assay conditions used to measure PEPc activity can influence the absolute values of the kinetic parameters.

### Assay conditions, extraction method, and source can affect PEPc kinetics

MIMS can directly measure dissolved CO_2_ even at very low C_i_ concentrations below the *K*_HCO3_ of PEPc (Beckmann *et al.*, 2009; Cousins *et al.*, 2010). Previously, Boyd *et al.* (2015) reported a MIMS-measured *K*_HCO3_ value of 62.8 µM for the *S*. *viridis* C_4_ PEPc which is higher than our MIMS-measured *K*_HCO3_ value of 26.6 µM for the *F. trinervia* C_4_ PEPc extracted from *E. coli*. This difference in *K*_HCO3_ between the *S. viridis* and *F. trinervia* C_4_ PEPc may be due to any combination of species differences in enzyme kinetics, enzyme purity, pH of the assay, and MIMS calibrations. Plant PEPc extracted from *F*. *trinervia*, a dicot in the Asteraceae family, and *S*. *viridis*, a monocot in the Poaceae family, had similar *K*_HCO3_ values at pH 7.6 (Fig. 5A). Bauwe (1986) also reported similar *K*_HCO3_ values for different C_4_ PEPc isoforms extracted from multiple grasses and *Glomphrena globosa*, a dicot from the Amaranthaceae family.

The *F. trinervia* C_4_ PEPc partially purified from *E. coli* was reported to be unphosphorylated at the N-terminal serine residue (Svensson *et al.*, 1997) and had a significantly lower *K*_HCO3_ than the desalted plant PEPc extracts taken from *F. trinervia* leaves during the day (Fig. 5). This suggests that potential differences in post-translational modifications might influence the kinetic properties of PEPc. Parvathi *et al.* (2000) observed a decrease in *K*_HCO3_ as PEPc changed from the unphosphorylated to the phosphorylated state, and that PEPc extracts from illuminated leaves had lower *K*_HCO3_ values than PEPc extracted in the dark. In the current study, the phosphorylation status of the PEPc extracts was not tested, so it cannot be confirmed that the difference in *K*_HCO3_ between the plant and *E*. *coli* extracts is due to changes in PTMs. Alternatively, Bauwe (1986) observed that unpurified C_4_ PEPc had higher *K*_HCO3_ values than purified C_4_ PEPc extracts. It is possible that the impurity of our desalted plant PEPc extracts from *F*. *trinervia* contributed to the increased *K*_HCO3_ relative to the C_4_ PEPc purified from *E*. *coli*. The potential differences in PTMs and enzyme purity do not completely explain why the *K*_HCO3_ for the *S. viridis* PEPc reported here and by Boyd *et al.* (2015) differ; however, this discrepancy in *K*_HCO3_ can be explained by differences in assay conditions and MIMS calibrations.

Raising the pH of the PEPc assay from 7.6 to 7.8, the pH used by Boyd *et al.* (2015), increased the *S. viridis* PEPc *K*_HCO3_ by ~21% (Fig. 5A, B). In addition to the pH of the assay buffer, the MIMS calibration method can alter the measured *K*_HCO3_. This is because two MIMS calibrations are required to convert a voltage signal of mass 44 to a micromolar concentration of CO_2_ and to determine the HCO_3_^−^ concentration in the reaction cuvette. Since the development of a novel MIMS technique to measure *K*_HCO3_ of PEPc (Boyd *et al.*, 2015), we have improved the MIMS calibration method to obtain more accurate *K*_HCO3_ values to analyze kinetic differences between PEPc isoforms. The calibration method presented here differed from that of Boyd *et al.* (2015) because all reaction components except the enzyme extract were included in the calibration. This would account for slight pH changes to the assay when adding DTT, G6-P, or PEP, which is important for determining the CO_2_:HCO_3_^−^ ratio. If there is a slight reduction in pH from adding assay components that is not accounted for during the calibrations, then the CO_2_:HCO_3_^−^ ratio can be slightly overestimated, leading to higher estimations of *K*_HCO3_. Using the calibration method and pH of 7.8 from Boyd *et al.* (2015), the *K*_HCO3_ values reported here (62.9 ± 8.7 µM) and by Boyd *et al.* (2015) (62.8 ± 5.0 µM) were nearly identical (Fig. 5B), suggesting that assay conditions such as pH and differences in MIMS calibration methods can affect the estimated *K*_HCO3_. These findings also highlight the important consideration of how well *in vitro* assay conditions reflect the *in vivo* conditions where PEPc operates. So far, *in vivo* PEPc kinetics can only be obtained by models using gas exchange (von Caemmerer, 2000). Further research is needed to compare *in vitro* and *in vivo* PEPc kinetics, since accurate PEPc kinetics are needed to model C_4_ photosynthesis.

### *K*_HCO3_ affects modeled rates of C_4_ photosynthesis

The C_4_ photosynthesis model developed by von Caemmerer (2000) was used to test if differences in *K*_HCO3_ between the C_3_ and C_4_ PEPc isoforms were enough to influence rates of net CO_2_ assimilation during C_4_ photosynthesis. The C_4_ model predicts that a lower *K*_HCO3_ may not affect photosynthetic rates under high CO_2_ partial pressures (Fig. 6). This is expected since C_4_ photosynthesis rates are typically not limited by PEPc under these conditions (von Caemmerer, 2000). However, a large *K*_HCO3_ may limit rates of C_4_ photosynthesis under low CO_2_ partial pressures (Fig. 6), for example when reduced stomatal conductance limits CO_2_ movement into the leaf. Flux control analysis found that PEPc has substantial control of C_4_ photosynthesis under low CO_2_ partial pressures (Dever *et al.*, 1997; Bailey *et al.*, 2000). Therefore, there is likely to be strong selective pressure to increase PEPc affinity for HCO_3_^−^ in C_4_ plants to increase the amount of C_i_ entering C_4_ photosynthesis. Our results, combined with those of others (Bläsing *et al.*, 2000; Engelmann *et al.*, 2002; Endo *et al.*, 2008), show that as C_4_*K*_HCO3_ dropped, there was a concurrent increase in C_4_*K*_PEP_. Due to the higher PEP levels observed in leaves of C_4_ plants compared with C_3_ plants (Leegood and von Caemmerer, 1994), it can be argued that there was stronger selective pressure to increase PEPc affinity for HCO_3_^−^ than to maintain high affinities for PEP since an increase in *K*_PEP_ may not negatively impact C_4_ photosynthesis rates to the extent that changes in *K*_HCO3_ can under lower CO_2_ partial pressures (Fig. 6).

### Conclusion

The direct comparison of closely related C_3_ and C_4_ PEPc isoforms from *Flaveria* demonstrates that the photosynthetic C_4_ PEPc isoform has a significantly higher affinity for HCO_3_^−^ than its closely related C_3_ PEPc isoform. This reduced *K*_HCO3_ impacts net CO_2_ assimilation rates, particularly at low CO_2_ availability, suggesting selective pressure to reduce the C_4_*K*_HCO3_ to optimize inorganic carbon flux through C_4_ photosynthesis. Alternatively, the increase in *K*_PEP_ can be seen as strengthening the diurnal regulation of C_4_ PEPc but residue S774 appears to link *K*_HCO3_ and *K*_PEP_, indicating that the increase in *K*_PEP_ could be a negative consequence of reducing *K*_HCO3_. Testing different plant species will provide new insights into which amino acids control *K*_HCO3_ and provide a better understanding of the structure and function relationship of the enzyme. Obtaining a better understanding of what controls *K*_HCO3_ will also lead to enhancing C_4_ photosynthesis, particularly at low CO_2_ partial pressures when stomata are partially closed. This raises interesting questions of whether there is a range in *K*_HCO3_ across the diverse lineages of C_4_ plants and finding relationships between certain amino acid residues and ranges of *K*_HCO3_ values can be beneficial for promoting strategies to optimize C_4_ photosynthesis in crop species for drought conditions.

## Supplementary data

Supplementary data are available at *JXB* online.

Table S1. Variable descriptions and values used to model C_4_ photosynthesis.

Table S2. Kinetic properties of PEPc isoforms from *F. trinervia* and *F. pringlei*.

Table S3. Kinetic properties of PEPc isoforms in the presence of 2.5 mM malate.

Fig. S1. Representative MIMS responses of the C_3_ and C_4_ PEPc activities with changing HCO_3_^−^ concentrations.

Fig. S2. HCO_3_^−^ response curves for the C_3_, C_3_-A774S, C_4_, and C_4_-S774A PEPc isoforms in the presence of 2.5 mM malate.

Supplement Data

## Abbreviations

CA: carbonic anhydrase
C_i_: inorganic carbon
C_m_: mesophyll CO_2_ concentration
G6-P: glucose 6-phosphate
h: Hill value
HCO_3_^−^: bicarbonate
*K*_HCO3_: *K*_m_ for HCO_3_^−^
*K*_PEP_: *K*_m_ for PEP
MIMS: membrane-inlet mass spectrometry
OAA: oxaloacetate
PEG: polyethylene glycol
PEP: phospho*enol*pyruvate
PEPc: phospho*enol*pyruvate carboxylase
PTM: post-translational modification
*V*_P_: PEPc activity
*V*_Pmax_: maximum rate of PEPc carboxylation
*V*_Pmax(plant)_: maximum rate of PEPc carboxylation per unit leaf area.
