# Supplementary material for: A single serine to alanine substitution decreases bicarbonate affinity of phosphoenolpyruvate carboxylase in C4Flaveria trinervia
Source: J Exp Bot. 2018 Dec 4;70(3):995–1004. doi: 10.1093/jxb/ery403 (PMC6363079; doi:10.1093/jxb/ery403)
Supplement: Supplement Data [file ery403_suppl_supplement_data.pdf]

SUPPLEMENTARY DATA

Table S1. Variable descriptions and values used to model C_4 photosynthesis. The following values were used in the von Caemmerer (2000) equations to model the influence of the C_3 and C_4 K_{HCO3} on rates of C_4 photosynthesis.

**Variable**	**Description**	**Value**	**Reference**
α	Fraction of PSII activity in the bundle sheath	0.2 (*F. bidentis*)	Ubierna *et al*. (2013)
γ_*	Half the reciprocal of Rubisco specificity	0.000193	von Caemmerer *et al*. (1994)
A_c	C_4 enzyme-limited photosynthetic CO_2 assimilation	Variable (µmol m^{-2} s^{-1})	von Caemmerer (2000)
C_m	Estimated mesophyll CO_2 concentration	Variable (Pa)	von Caemmerer (2000)
g_{bs}	Bundle sheath conductance to CO_2	3 mmol m^{-2} s^{-1}	von Caemmerer (2000)
K_C	K_m (CO_2) for Rubisco	65 Pa	von Caemmerer (2000)
K_{HCO3}	K_m (HCO_3^-) for PEPc	6.5 or 15.9 Pa CO_2[a]	present work
K_O	K_m (O_2) for Rubisco	45,000 Pa	von Caemmerer (2000)
O_m	Estimated mesophyll O_2 concentration	21,000 Pa, assumed	Tholen and Zhu (2011)
R_d	Mitochondrial respiration in the leaf	$0.01V_{Cmax}$	von Caemmerer (2000)
R_m	Mitochondrial respiration in the mesophyll	$0.5R_d$	von Caemmerer (2000)
V_{Cmax}	Rubisco carboxylation maximum rate	60 µmol m^{-2} s^{-1}	von Caemmerer (2000)
V_P	PEP carboxylation	Variable (µmol m^{-2} s^{-1})	von Caemmerer (2000)
$V_{Pmax(plant)}$	Maximum rate of PEPc per unit leaf area	120 µmol m^{-2} s^{-1}	von Caemmerer (2000)
V_{pr}	PEP regeneration rate	80 µmol m^{-2} s^{-1}	von Caemmerer (2000)

[a]A pK_a of 6.12 and assumed mesophyll cytosol pH of 7.2 were used to convert µM HCO_3^- to µM CO_2. Pa CO_2 was obtained by using Henry's constant for CO_2 (0.034 mol L^{-1} atm^{-1}) and assumed standard pressure (101,325 Pa atm^{-1}).

$$a = 1 - \frac{\alpha}{0.047}\frac{K_C}{K_O} \quad \text{(Eqn S1)}$$

$$b = -\{(V_P - R_m + g_{bs}C_m) + (V_{Cmax} - R_d) + g_{bs}(K_c(1 + O_m/K_O)) + \frac{\alpha}{0.047}(\gamma_* V_{Cmax} + (R_d K_C/K_O))\}$$ (Eqn S2)

$$c = (V_{Cmax} - R_d)(V_P - R_m + g_{bs}C_m) - (V_{Cmax}g_{bs}\gamma_* O_m + (R_d g_{bs} K_C(1 + \frac{O_m}{K_O}))$$ (Eqn S3)

Equations S1, S2, and S3 were input into the quadratic, Eqn S4, to solve for Ac.

$$A_c = \frac{-b - \sqrt{b^2 - 4ac}}{2a}$$ (Eqn S4)

To insert K_{HCO3} as a variable into the C_4 model, the Michaelis-Menten equation was substituted for V_P in equations S2 and S3:

$$V_P = \frac{V_{Pmax} * [HCO_3^-]}{K_{HCO3} + [HCO_3^-]}$$ (Eqn S5)

Once PEP regeneration becomes limiting, V_P equals V_{pr} and V_P is held constant at 80 µmol m^{-2} s^{-1} (von Caemmerer, 2000). The Michaelis-Menten equation was used instead of the Hill equation since the Hill value was essentially 1.0.

Table S2. Kinetic properties different PEPc isoforms. Assays were conducted using 100 mM HEPES-KOH buffer (pH 7.6) in the presence or absence of 5 mM G6-P. Kinetic parameters were solved using the Hill Equation and values represent the mean ± standard deviation of four independent PEPc samples extracted from *E. coli*. A two-way ANOVA test determined G6-P had no effect on V_{Pmax}, K_{HCO3}, and Hill values. Tukey HSD tests were used to determine significant differences in V_{Pmax}, K_{HCO3}, and Hill values between the different PEPc isoforms. Hill values were not significantly different from one another. Different letters represent significant differences ($P < 0.005$).

**Isoform**	**V_{Pmax} (µmol mg protein^{-1} min^{-1})**		**K_{HCO3} (µM)**		**Hill Value (*h*)**	
	**(+) G6-P**	**(-) G6-P**	**(+) G6-P**	**(-) G6-P**	**(+) G6-P**	**(-) G6-P**
C_3	5.1 ± 0.7[b]	4.4 ± 0.6[b]	64.0 ± 2.4[a]	57.6 ± 2.1[a]	1.0 ± 0.1[a]	1.0 ± 0.1[a]
C_3-A774S	5.7 ± 1.7[b]	5.1 ± 1.5[b]	61.5 ± 9.1[a]	65.1 ± 9.2[a]	1.0 ± 0.1[a]	1.0 ± 0.1[a]
C_4	8.1 ± 0.7[a]	7.3 ± 0.4[a]	26.6 ± 1.7[c]	25.9 ± 3.2[c]	1.0 ± 0.1[a]	1.0 ± 0.1[a]
C_4-S774A	7.7 ± 1.3[a]	7.8 ± 1.1[a]	38.6 ± 5.5[b]	38.4 ± 5.7[b]	1.0 ± 0.1[a]	1.0 ± 0.1[a]

Table S3. Kinetic properties of different PEPc isoforms in the presence of 2.5 mM malate. Assays were conducted using 100 mM HEPES-KOH buffer (pH 7.6) in the presence or absence of 5 mM G6-P. Kinetic parameters were solved using the Hill Equation. Values represent the mean ± standard deviation of four independent PEPc samples extracted from *E. coli*. A two-way ANOVA test determined G6-P had no effect on V_{Pmax}, K_{HCO3}, and Hill values. Different letters represent significant differences ($P < 0.01$).

**Construct**	**V_{Pmax} (µmol mg protein^{-1} min^{-1})**		**K_{HCO3} (µM)**		**Hill Value (*h*)**	
	**(+) G6-P**	**(-) G6-P**	**(+) G6-P**	**(-) G6-P**	**(+) G6-P**	**(-) G6-P**
FP966 (C_3)	5.0 ± 0.7[a]	4.1 ± 0.5[a]	68.3 ± 4.9[a]	65.8 ± 14.2[a]	1.0 ± 0.1[a]	1.0 ± 0.1[a]
FP966-A774S	4.9 ± 1.2[a]	N/A	67.0 ± 11.2[a]	N/A	1.1 ± 0.1[ab]	N/A
FT966 (C_4)	4.5 ± 0.8[ac]	3.1 ± 1.2[ac]	36.5 ± 7.3[b]	30.7 ± 1.7[b]	0.9 ± 0.1[a]	1.0 ± 0.1[a]
FT966-S774A	5.5 ± 1.0[ab]	5.9 ± 0.8[ab]	40.7 ± 5.6[b]	48.1 ± 9.5[b]	0.9 ± 0.1[ac]	0.9 ± 0.1[a]

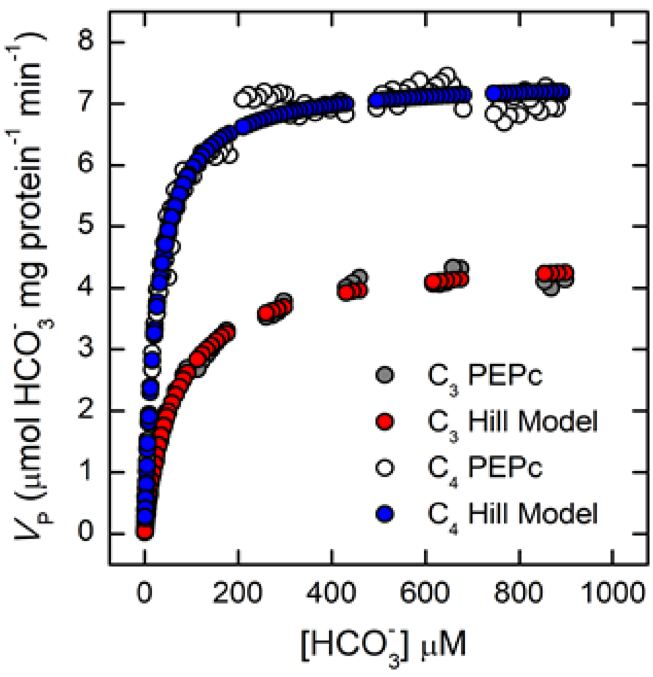

Figure S1. Representative response of the C_3 and C_4 PEPc activities with changing HCO_3^- concentrations. The values for V_{Pmax}, K_{HCO3}, and *h* were solved by fitting the Hill Equation (closed blue and red circles) to the C_4 PEPc (open circles) and C_3 PEPc (closed circles) response to HCO_3^- from the MIMS. These PEPc assays were conducted in 100 mM HEPES-KOH (pH 7.6), 10 mM $MgCl_2$, 1 mM DTT, 50 µg mL^{-1} CA, 5 mM G6-P, 5 mM PEP, and various HCO_3^- concentrations. Each HCO_3^- response curve is a single representation of the C_3 and C_4 PEPc isoforms.

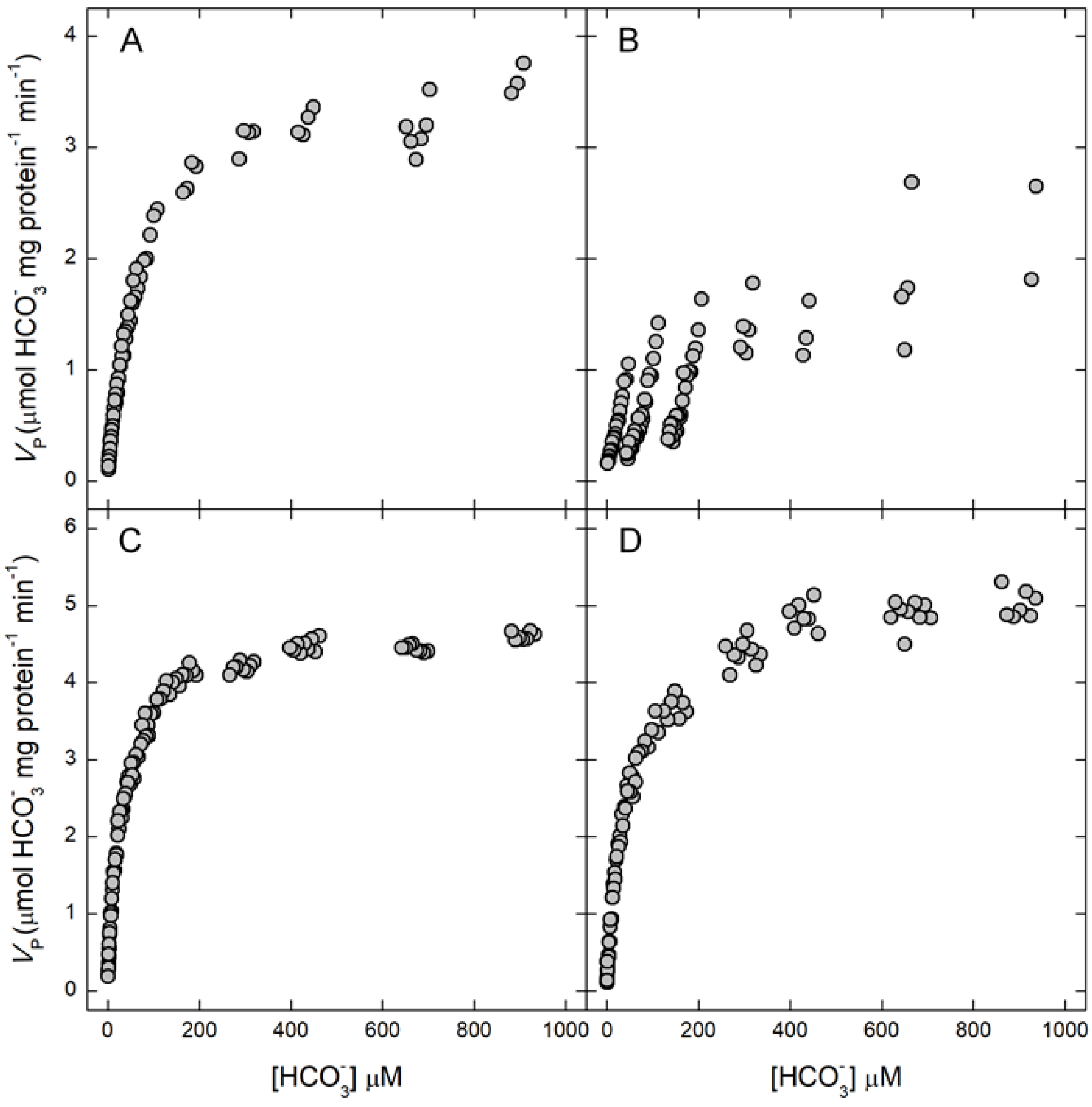

Figure S2. HCO_3^- response curves for the (A) C_3, (B) C_3-A774S, (C) C_4, and (D) C_4-S774A PEPc isoforms in the presence of 2.5 mM malate. The C_3-A774S PEPc was unable to produce a typical Michaelis-Menten substrate response curve to determine reliable V_{Pmax}, K_{HCO3}, and Hill values due to the severe malate inhibition exhibited. HCO_3^- response PEPc assays were conducted in 100 mM HEPES-KOH (pH 7.6), 10 mM $MgCl_2$, 1 mM DTT, 50 μg mL^{-1} CA, 2.5 mM malate

(pH 7.6), 5 mM PEP, and various HCO_3^- concentrations. Each HCO_3^- response curve is a single representation for each PEPc isoform.

Supplementary Material References

Tholen D, Zhu X-G. 2011. The mechanistic basis of internal conductance: a theoretical analysis of mesophyll cell photosynthesis and CO_2 diffusion. Plant Physiology **156**, 90–105.

Ubierna N, Sun W, Kramer DM, Cousins AB. 2013. The efficiency of C_4 photosynthesis under low light conditions in *Zea mays*, *Miscanthus* x *giganteus* and *Flaveria bidentis*. Plant, Cell & Environment **36**, 365–381.

von Caemmerer, S. (2000) *Biochemical Models of Leaf Photosynthesis*, CSIRO Publishing, Collingwood VIC, Australia.

von Caemmerer S, Evans JR, Hudson GS, Andrews TJ. 1994. The kinetics of ribulose-1,5-bisphosphate carboxylase/oxygenase in vivo inferred from measurements of photosynthesis in leaves of transgenic tobacco. Planta **195**, 88–97.
